# Regional trade and the nutrition transition: opportunities to strengthen NCD prevention policy in the Southern African Development Community

**DOI:** 10.3402/gha.v8.28338

**Published:** 2015-07-22

**Authors:** Anne Marie Thow, David Sanders, Eliza Drury, Thandi Puoane, Syeda N. Chowdhury, Lungiswa Tsolekile, Joel Negin

**Affiliations:** 1 Menzies Centre for Health Policy, University of Sydney, Sydney, Australia; 2 School of Public Health, University of the Western Cape, Cape Town, South Africa; 3 School of Public Health, University of Sydney, Sydney, Australia

**Keywords:** trade, Southern Africa, nutrition, health, food

## Abstract

**Background:**

Addressing diet-related non-communicable diseases (NCDs) will require a multisectoral policy approach that includes the food supply and trade, but implementing effective policies has proved challenging. The Southern African Development Community (SADC) has experienced significant trade and economic liberalization over the past decade; at the same time, the nutrition transition has progressed rapidly in the region. This analysis considers the relationship between regional trade liberalization and changes in the food environment associated with poor diets and NCDs, with the aim of identifying feasible and proactive policy responses to support healthy diets.

**Design:**

Changes in trade and investment policy for the SADC were documented and compared with time-series graphs of import data for soft drinks and snack foods to assess changes in imports and source country in relation to trade and investment liberalization. Our analysis focuses on regional trade flows.

**Results:**

Diets and the burden of disease in the SADC have changed since the 1990s in parallel with trade and investment liberalization. Imports of soft drinks increased by 76% into SADC countries between 1995 and 2010, and processed snack foods by 83%. South Africa acts as a regional trade and investment hub; it is the major source of imports and investment related to these products into other SADC countries. At the same time, imports of processed foods and soft drinks from outside the region – largely from Asia and the Middle East – are increasing at a dramatic rate with soft drink imports growing by almost 1,200% and processed snack foods by 750%.

**Conclusions:**

There is significant intra-regional trade in products associated with the nutrition transition; however, growing extra-regional trade means that countries face new pressures in implementing strong policies to prevent the increasing burden of diet-related NCDs. Implementation of a regional nutrition policy framework could complement the SADC's ongoing commitment to regional trade policy.

The World Health Organization's Global Action Plan for the Prevention and Control of NonCommunicable Diseases (NCDs) was endorsed in 2013 and draws attention to the growing burden of diet-related NCDs in low- and middle-income countries (1). While much of the focus of the global community is on individual consumption as a key factor in diet-related NCD prevention, the food environment is also an important determinant of what foods are available to the consumer (2–4). The NCD Action Plan recommends policy interventions such as labeling, fiscal measures, and advertising restrictions to complement nutrition education for consumers.

African countries continue to battle high rates of infectious disease and malnutrition; however, rising rates of NCDs are causing significant concern (5, 6). This emerging NCD epidemic is associated with significant social, economic, and personal costs due to changing health care needs and lost productivity (7, 8). Risk factors for diet-related NCDs are becoming increasingly prevalent, pointing to an urgent need for prevention (Fig. 1).

**Fig. 1:**
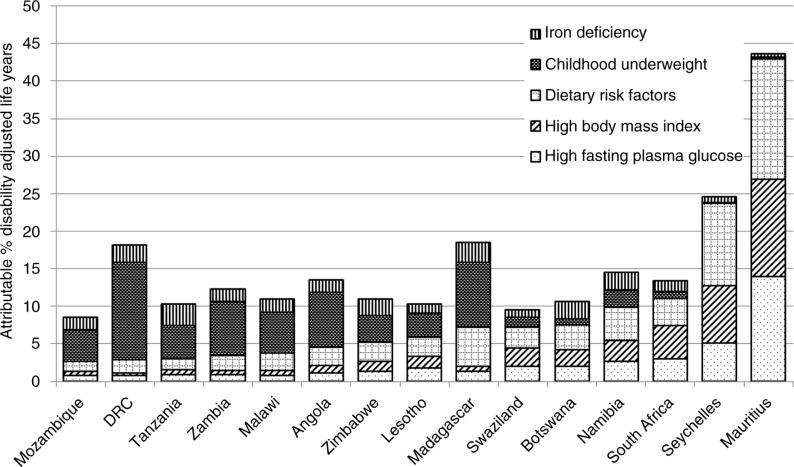
Nutrition related risk factors in the SADC countries and their contribution to the total burden of disability adjusted life years. Global Burden of Disease Project 2010, available at: www.healthmetricsandevaluation.org/gbd/visualizations/gbd-cause-patterns Note: ‘Dietary risk factors’ includes: diet low in nuts and seeds, diet low in fruits, diet low in seafood omega-3 fatty acids, diet low in whole grains, diet high in sodium, diet high in processed meat, diet low in vegetables, diet low in fiber, diet low in polyunsaturated fatty acids, diet high in trans fatty acids, and diet high in sugar-sweetened beverages.

This epidemiological transition has been associated with concurrent nutrition transition. While rates of undernutrition remain high, populations are shifting away from traditional (high nutrient and fibrous) staple foods to consume more highly refined, often imported, cereals, processed and packaged foods, cooking oils, and processed meats; dietary patterns associated with increased risk of diet-related disease (9–11).

A key dietary change in Southern African Development Community (SADC) countries associated with the nutrition transition has been increasing intakes of processed snack foods and soft drinks, which contain dietary calories but few other nutrients, and can displace nutrient-dense foods from the diet. Diets high in salt, saturated and trans fats, and sugar – including through consumption of highly processed foods and sugar-sweetened soft drinks – are associated with overweight and obesity (itself a risk factor for a wide range of NCDs), as well as cancer, diabetes, and cardiovascular diseases (12–19). Epidemiological studies have documented increased consumption of processed snack foods and sugary beverages in all SADC countries since the mid-1990s, along with decreased consumption of healthy traditional foods (6, 20–23). This change has occurred over the same period that the food environment has changed substantially, with highly processed food and soft drinks becoming more affordable and accessible (10, 11). For example, the market for soft drinks in South Africa more than doubled from 2,294 million liters in 1998 to 4,746 million liters in 2012, and over the same period the value of the market for packaged food increased substantially in real terms, from 55,815 million Rand to 61,286 million Rand (143,050 million Rand in 2012 dollars, adjusted for average inflation to 1998 dollar equivalent) (24, 25).

Globally, it is clear that trade and foreign direct investment (FDI) liberalization policies have had a complex effect on food environments and nutrition, contributing to increased availability and consumption of refined and processed foods that are linked to NCDs, and decreased availability of traditional staple foods (26). There is thus growing interest in the use of trade policy to improve diets and prevent NCDs (27).

In this article, we consider the role of trade as an ‘upstream’ determinant of food availability and the changes in diet and NCD prevalence that have occurred in the SADC. The SADC – comprised of 15 countries, 277 million people, and a GDP of US$575 billion (Table 1) – has taken a proactive approach to regional trade liberalization, with a strong regional agenda to pursue liberalization (28, 29). South Africa acts as a trade hub in the region, with the largest economy and the second highest population: two-thirds of total SADC trade is with South Africa (30), with a particular dependence on South Africa as a source of imports (29). South Africa also has the most diversified production base in the region, with a strong domestic food processing sector, while many of the other SADC countries are dependent on a limited number of primary commodity exports (namely mineral, agricultural, or petroleum-based commodities) (29). South Africa has also received by far the most FDI in the region (Table 1).

**Table 1:** Overview of key economic statistics for the Southern African Development Community

	Population (total in millions)		GDP per capita (current US$)		Exports of goods and services (% of GDP)		Imports of goods and services (% of GDP)		FDI Inflows, (US$ billion)
					
SADC member country	2000	2012	2000	2012	2000	2012	2000	2012	2008
Angola	13.9	20.8	656	5,539	90	59	63	42	1.7
Botswana	1.7	2.0	3,297	7,255	52	43	40	59	0.1
Congo, Dem. Rep.	46.9	65.7	407	418	11	NA	16	NA	1.0
Lesotho	1.9	2.1	415	1,135	35	49	135	114	0.2
Madagascar	15.7	22.3	246	443	31	29	38	39	1.5
Malawi	11.3	15.9	154	267	26	NA	35	NA	0.0
Mauritius	1.2	1.3	3,861	8,862	61	55	62	67	0.4
Mozambique	18.2	25.2	236	570	16	30	37	71	0.6
Namibia	1.9	2.3	2,059	5,931	41	44	45	54	0.5
Seychelles	0.8	0.9	7,579	11,689	32	NA	55	NA	0.4
South Africa	44.0	52.3	3,020	7,314	28	30	25	32	9.6
Swaziland	1.1	1.2	1,433	3,290	74	NA	88	NA	0.0
Tanzania	34.0	47.8	308	609	13	30	20	47	0.7
Zambia	10.1	14.1	322	1,463	26	46	40	43	0.9
Zimbabwe	12.5	13.7	535	909	38	33	36	61	0.1

Source: World Bank Development Indicators (www.databank.worldbank.org/data/); Investment data only: Southern African Development Community (www.sadc.int/themes/economic-development/investment/foreign-direct-investment/). Note: detailed investment data are not available via the World Bank Development Indicators. NA: Data not available.

This research aims to identify levers that could help to ensure that proactive trade and investment policy in the region is also matched by proactive public health policies for NCD prevention. From a public health perspective, the food environment is under-researched in this region. We know little about where processed and packaged food in the region is coming from, and the potential role of trade and investment in increasing availability. While there are likely to be complex and differential effects on the availability of healthy and unhealthy foods due to trade and investment [e.g. (31, 32)] – such as multiple probable effects on fruit and vegetable availability due to growth in imports and rising opportunities for exports – examining trade in processed and packaged food provides timely insights into a key aspect of nutrition transition in the region and may pave the way for further research in this area.

## Methods

### Data

This analysis takes a regional approach to understanding availability of unhealthy foods as an upstream driver of poor diets, with the aim of identifying strategies to strengthen NCD prevention policy. This policy analysis utilizes food trade data from the Food and Agriculture Organization's FAOSTAT database (33) for all countries in the SADC for the period 1996–2010, for non-alcoholic beverages (excluding fruit juice) and snack foods (ice cream, sugar confectionery, wafers, chocolate products, popcorn, and pastry – including all baked products other than bread – i.e. cakes, biscuits etc.). FDI data were obtained from Euromonitor International and the World Trade Centre Investment map.

Information on regional and country-level trade and investment policies was obtained from World Trade Organization (WTO) trade policy reviews, the South Africa Department of Trade, and a review of academic literature. While this analysis is limited by the partial data and policy information on trade and FDI that are available for this region (34), the data used are the best currently available, and have been the basis for similar studies (e.g. 31, 32). The analysis also aligns with the INFORMAS approach to monitoring the impact of trade agreements on nutrition (35).

### Analysis

Data on changes in import volume and source country for non-alcoholic beverages (soft drinks) and snack foods were analyzed over time (using Microsoft Excel), and compared with changes in trade and investment policy in the SADC, to identify patterns at a regional level. The analysis is presented by source country of imports, focused on intra-regional compared to extra-regional trade flows. South Africa is considered separately as the largest economy in the region and a recognized regional trade hub (36).

## Results

### Regional trade and investment policy in the SADC

The SADC is one of the most proactive regional trade blocs in the developing world, and has consistently supported policies to bring down trade barriers between countries in the region (See timeline in Box 1). SADC trade is dominated by South Africa, which is the largest economy in the region and has actively supported domestic and regional liberalization since the end of apartheid in 1994 (36, 37). South Africa has instituted specific policies to liberalize trade, attract investment, and support regional export and investment within the SADC (from South Africa into the other SADC countries) (38). South Africa has also been active in promoting its extra-regional trade and investment capacity, signing 22 Bilateral Investment Agreements between 1997 and 2003, and also a Bilateral Trade Agreement with the European Union (EU) (Box 1).

This intra- and extra-regional liberalization has been reflected by the SADC more broadly. In addition to instituting a regional Free Trade Area in 2008, the SADC has focused on macro-economic policy stability and harmonization among member countries, which also serves to make the region more attractive to extra-regional trade and investment (29, 39). All SADC members are also members of the WTO, and have implemented extra-regional liberalization measures in line with WTO commitments.

***Box 1*. Major regional trade and investment policy directions in the SADC since 1990**

Early 1990s: ongoing liberalization associated with multilateral trade negotiations

1996: SADC trade agreement signed

1997–2003: South Africa strengthens investment policy and signs 22 Bilateral Investment Agreements

1999: South Africa signs bilateral trade agreement with European Union (EU)

2000: SADC trade protocol comes into effect; Government of South Africa strengthens support for regional export and investment

2002: new Southern Africa Customs Union Agreement completed

2007: Interim Economic Partnership Agreement concluded between EU and Botswana, Lesotho, Namibia, Swaziland, and Mozambique

2008: SADC Free Trade Area completed (except for Angola, Democratic Republic of the Congo, and Seychelles)

Sources: (28, 34, 36, 38, 40).

### Trade in processed food and sugary drinks

**Fig. 2:**
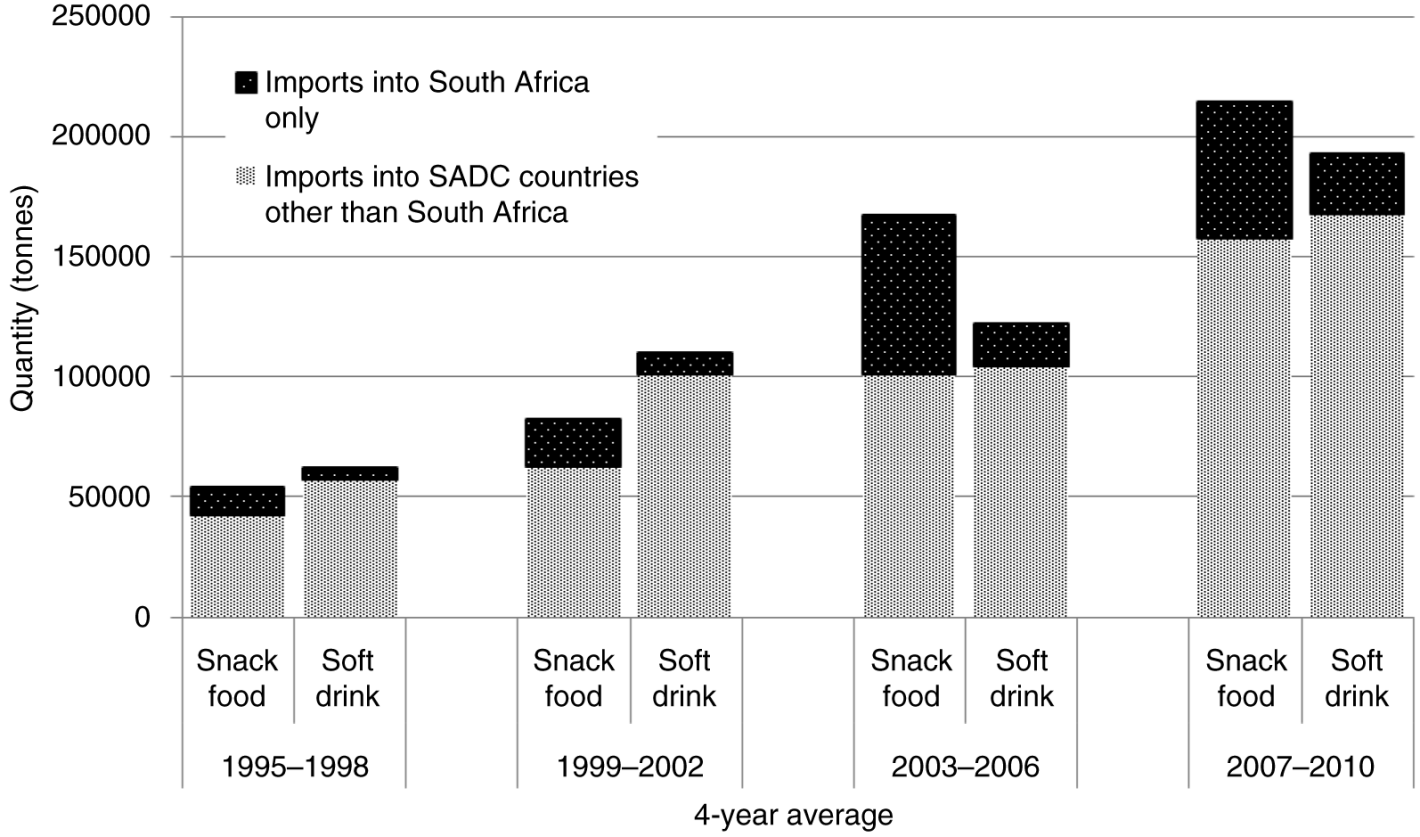
Total imports of soft drinks and processed snack foods into South Africa and other SADC countries. FAOSTAT detailed trade data (16). Notes: ‘Soft drinks’ refers to non-alcoholic beverages excluding fruit juice. Snack food categories: ice cream, sugar confectionery, wafers, pastry (this includes all baked products other than bread – i.e. cakes, biscuits etc.), chocolate products, and popcorn.

Total imports of processed snack foods and soft drinks into South Africa and the other SADC countries increased markedly between 1995 and 2010 (Fig. 2, Table 2). Imports of soft drinks increased by 92% into South Africa and 76% into the other SADC countries, with the largest growth in 2003–2006 and 2007–2010, despite the global financial crisis that occurred in the latter period (Fig. 2). This likely reflects the implementation of the EU-South Africa Agreement in 1999, which increased imports into South Africa considerably (36), and subsequently the implementation of the regional Free Trade Area in 2008, with its accompanying common external tariff and efforts to increase macro-economic policy stability (29, 39); there was a 45% increase in soft drink imports into SADC countries other than South Africa between 2007 and 2008 alone.

**Table 2:** Imports of soft drinks and snack foods into SADC countries (other than South Africa) by source, 3-year average

	Imports from South Africa	Imports from other SADC countries	Imports from countries outside the region	Total	Imports from South Africa	Imports from other SADC countries	Imports from countries outside the region	Total
		
	Total trade volumes (metric tonnes)				% of imports by source			
**Processed snack foods**								
1996–1998	15,586	1,505	2,467	19,558	80	8	12	100
2002–2004	21,908	2,307	9,579	33,794	65	7	28	100
2008–2010	24,689	6,388	18,522	49,599	50	13	37	100
**Soft drinks**								
1996–1998	12,779	4,661	1,408	18,848	68	25	7	100
2002–2004	12,622	7,591	4,837	25,050	50	30	20	100
2008–2010	24,921	7,969	16,713	49,604	50	16	34	100

Source: FAOSTAT detailed trade matrix (33). Data missing for Angola, Lesotho, and Mozambique; 2004–2006 data for Botswana, Namibia, Malawi, DRC missing; 2006–2007 data for Zimbabwe missing. Snack food categories: ice cream, sugar confectionery, wafers, pastry (this includes all baked products other than bread – i.e. cakes, biscuits), and chocolate products (popcorn data not available in detailed trade matrix). ‘Soft drinks’ refers to non-alcoholic beverages excluding fruit juice.

Imports of processed snack foods increased by 83% into both South Africa and the other SADC countries between 1995 and 2010, with significant growth occurring in imports to South Africa after the signing of the EU agreement (Fig. 2). Similar to the situation with soft drinks, the largest increase in imports of snack foods into the other SADC countries occurred after the implementation of the regional Free Trade Area in 2008 (there was a nearly 30% increase between 2007 and 2008 alone).

### Regional dynamics in soft drink and processed snack food trade

As imports have increased, FDI in soft drinks and processed snack foods in the region has also grown – mainly to South Africa, which has been the major recipient of investment in the SADC (41). While South Africa does import processed foods and beverages for domestic consumption, this FDI means it also acts as a regional hub for processed, packaged food, and soft drink manufacturing and subsequent export to SADC countries. Growth in inward FDI has enabled South Africa to expand its domestic food processing industries and retain its dominance in regional exports in the face of increasing imports from outside of the region.

Current major multinational food industry investors in South Africa include The Coca-Cola Company, Unilever, Nestle, Parmalat, Cadbury Schweppes, PepsiCo Inc., Kellogg Company, Kraft Foods Inc., Mars Incorporated, ConAgra, and SAB Miller, establishing production of processed dairy products, soft drinks, snacks, and confectionery (42). The leading franchises for ‘fast’ food include KFC and McDonalds, as well as numerous local franchises, many of which are expanding throughout the region (25).

**Fig. 3:**
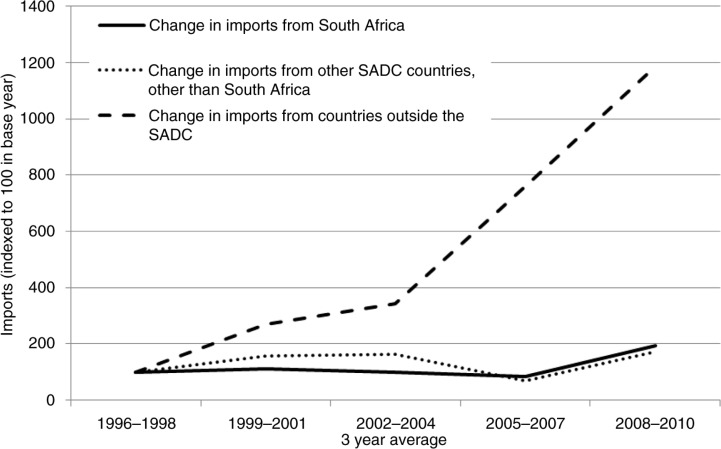
Growth of imports of soft drinks into SADC countries other than South Africa (indexed so that 1996–1998=100), by source. FAOSTAT detailed trade matrix (16). Notes: Data missing for Angola, Lesotho, and Mozambique; 2004–2006 data for Botswana, Namibia, Malawi, DRC missing; 2006–2007 data for Zimbabwe missing. ‘Soft drinks’ refers to non-alcoholic beverages excluding fruit juice.

In line with its significant production capacity, South Africa is the major source of soft drink imports into the other countries in the region: 50% of regional imports came from South Africa in 2008–2010 (Table 2). The volume of soft drink imports from South Africa into the other SADC countries rose sharply after the implementation of the Free Trade Area, with a doubling of volume imported between the period 1996–1998 and 2008–2010 (Fig. 3). This has varied between countries; for example, export data (available for only six destination countries) show that between 1997 and 2010, the volume of soft drinks exported from South Africa to Tanzania and Zimbabwe increased by over 4,000%, and by over 500% to Zambia and the Democratic Republic of the Congo (DRC) (33).

**Fig. 4:**
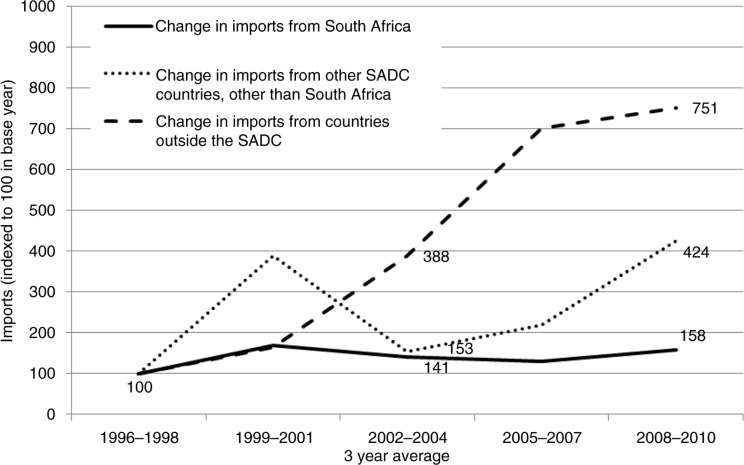
Growth of imports of processed snack foods into SADC countries other than South Africa (indexed so that 1996–1998=100), by source. FAOSTAT detailed trade matrix (16). Notes: Data missing for Angola, Lesotho, and Mozambique. Snack food categories: ice cream, sugar confectionery, wafers, pastry (this includes all baked products other than bread – i.e. cakes, biscuits), and chocolate products (popcorn data not available in detailed trade matrix).

South Africa is also the major source of processed snack food imports into the other countries in the region. In 1996–1998, 80% of total snack food imports were from South Africa (Table 2), and between 1996 and 2010 these imports grew by 150% (Fig. 4). For six countries the growth was much higher: export data (available for only a limited number of destination countries) show that between 1997 and 2010 processed snack food exports from South Africa to Tanzania, Malawi, and Zimbabwe increased by over 400%, and by over 200% to Zambia and Angola (33).

In addition to the high volume of within-SADC trade, there has been a flood of soft drink and snack food imports from outside the region in recent years. From 1996–1998 to 2008–2010 soft drink imports from outside the region grew by almost 1,200%, and processed snack food imports by 750% (Figs. 3 and 4). By 2008–2010, imports from outside the region into SADC countries constituted one-third of total soft drink imports [mainly from the EU, China, and the United Arab Emirates (UAE) (33)] and 40% of processed snack food imports [mainly from the EU, China, India, Brazil, and the UAE (33)] (Table 2). These extra-regional products are consistently cheaper than those produced locally (11).

FDI policies have also facilitated dramatic regional expansion by South African supermarkets, food processors, and the fast food sector (25, 41, 43). Expansion of formal food retail has contributed to increased accessibility and affordability of processed foods and beverages as a result of improved supply chains: prices of processed food products are cheaper in chain supermarkets compared to local stores in the region (44). By 2009, all of the 12 major South African supermarkets invested in at least one of the SADC countries (with all SADC countries receiving investment), and over 80% of all processed food products in Botswana, Namibia and Zambia were imported from South Africa (45).

## Discussion

This analysis shows that the nutritional and epidemiological transitions currently underway in Southern Africa have been accompanied by a dramatic change in the food environment, with increased access to processed foods and soft drinks within SADC facilitated by growing intra- and extra-regional trade and investment. The source of foods and beverages is changing: while South Africa retains its historical role as the regional trade hub, there has been a dramatic increase of highly processed foods and soft drinks entering Southern Africa from outside the region. This flood of low nutritional value foods at an early stage of the NCD epidemic highlights the need to support and enable smaller and vulnerable countries to establish strong nutrition policies for NCD prevention recommended by the World Health Organization (WHO), such as labeling, fiscal measures, and advertising restrictions.

While increasing trade and investment can contribute to economic growth, policy makers need to be aware of the high social and economic costs of NCDs: the SADC cannot afford its emerging NCD epidemic (7). The region needs strong, proactive, public health nutrition policy to avert this emerging epidemic. The fact that it is still at a relatively early stage means there is potential to minimize the growth of NCDs in the region through prevention. By examining the regional dynamics of the supply of soft drinks and processed snack foods in the SADC, two key products associated with nutrition transition, this study indicates the need to tackle prevention of diet-related NCDs not just at a country level, but also through a concerted regional approach. However, it should be noted that the analysis is limited by a lack of causality attribution and causal inference, and by the availability of detailed data only on trade in processed snack foods and soft drinks (not production or consumption) in the dataset. While our analysis shows a correlation between liberalization and increased trade in processed foods and soft drinks, flow on implications for consumption will also be affected by other factors, such as rising incomes or changes in pricing. Thus, these policy recommendations should be situated – as the WHO NCD Action Plan recommends – within broader, comprehensive policy action to improve the food supply and dietary choices at a country level.

### Policy implications

This analysis of trends and regional trade patterns in soft drinks and processed snack foods highlights two opportunities for regional policy action to stem the flood of high calorie and nutrient-poor processed foods, snacks, and beverages into the region, and thus contribute to reducing obesity and diet-related NCDs.

First, the importance of South Africa as a source of traded food indicates that food policies adopted by South Africa will shape the regional food supply, and there is thus scope for South Africa to lead NCD prevention in the region. As such, recent efforts by South Africa to set targets for salt reduction in foods may have positive regional repercussions, and its continued leadership in implementing the WHO NCD Global Action Plan will improve the food supply in the region.

However, to continue to strengthen food policy will require South African policy makers to address the implicit tension between good food policy on one hand and its quest to increase regional trade and attract investment on the other, as the latter reinforces industry intentions to increase regional markets for less healthy commodities (3). Similarly, in other countries within the SADC, the growing intra- and extra-regional trade volumes in less healthy products observed in this study means that pressure from trading partners could result in tension for country-level policy makers (46, 47). While increasing trade and investment remains a priority, and is supported by regional infrastructure through the SADC, strong nutrition policies have limited support at the regional level.

This leads to the second policy opportunity: a regional framework that supports countries to implement the food policy options of the WHO NCD Global Action Plan. Embedding a regional framework for NCD prevention, based on the WHO Global Action Plan, within the remit of a regional trade body such as the SADC, could improve policy coherence between trade and nutrition by enabling systematic consideration of nutrition policy objectives when potential tensions between trade and health policy arise. Such a framework would provide tools and capacity-building to strengthen NCD prevention policies in vulnerable countries within the region – particularly those at an early stage of the nutrition transition where there is significant potential for pre-emptive action to forestall an NCD epidemic.

Adopting such a regional approach is likely to be made easier by the SADC's existing commitment to food security, and the fact that the SADC cites the EU, which has adopted regional nutrition policies, as a model for regional integration in its trade policy and treaty making (48, 49). Trade policy makers in the SADC are thus likely to be familiar with EU food policies, and there may be scope to learn from EU regional policies on food labeling, health and nutrition claims, and revision of school feeding programs by provision of fruit and milk (linked to regional agricultural policies) (50–52). The SADC might also consider an advisory group to support regional NCD prevention policy, similar to the EU High Level Group on Nutrition and Physical Activity.

There is also scope to draw inspiration from innovation in the wider African region and facilitate learning between countries regarding best-practice interventions to prevent diet-related NCDs. For example, Ghana's innovative use of food standards to reduce availability of fatty meat (53) and South Africa's mandatory salt targets (54) could inform policy action in other countries. Developing a regional framework will also require engagement with the private sector. The regional framework could thus also establish best-practice approaches to industry engagement, which could minimize the potential for influence by vested interests (55).

## Conclusions

There is significant potential for strong nutrition policy to limit the extent of the NCD epidemic that is currently building in Southern Africa. However, such action may create tensions with established regional trade and investment policy, as trade and investment related to commodities associated with the nutrition transition is affected. Taking a proactive approach to improving policy coherence between trade and public health at the regional level would help to strengthen national nutrition policy making.
